# Priority research questions in microbiome-integrated urban design

**DOI:** 10.1128/msphere.00619-25

**Published:** 2025-10-14

**Authors:** Richard Beckett, Lorraine Archer, Alexia Barrable, Michael Bogdan-Margineanu, Sean Bradley, Sarah Hawes, Christianne Herr, Mira Housen, Alexandra Lacatusu, Olli Laitinen, Marja Roslund, Heather Rumble, William Scott, Aki Sinkkonen, Xin Sun, Jake M. Robinson

**Affiliations:** 1 The Bartlett School of Architecture, University College London 392549, London, United Kingdom; 2 Department of Plant Sciences, University of Cambridge 2152, Cambridge, England, United Kingdom; 3 Division of Psychology, Sociology and Education, Queen Margaret University, Edinburgh, Scotland, United Kingdom; 4 Cancer Neuroscience Laboratory, The Francis Crick Institute 376570, London, United Kingdom; 5 Faculty of Medicine, "Victor Babes" University of Medicine and Pharmacy, Timisoara, Romania; 6 Groundwork, London, United Kingdom; 7 College Green, Bristol City Council, The Council House 1988, Bristol, United Kingdom; 8 School of Design, SUSTech (Southern University of Science and Technology) 255310, Shenzhen, China; 9 Institute for Advanced Architecture of Catalonia, Carrer de Pujades, Barcelona, Spain; 10 Bio-ID Lab, The Bartlett School of Architecture, University College London 392549, London, United Kingdom; 11 Faculty of Medicine and Health Technology, Tampere University 101287, Tampere, Finland; 12 Natural Resources Institute Finland (Luke) 419837, Helsinki, Finland; 13 Centre for Sustainable Planning and Environments, University of the West of England (Bristol) 215786 , Bristol, United Kingdom; 14 Department of Microbial Diseases, UCL Eastman Dental Institute, University College London 15584, London, United Kingdom; 15 Natural Resources Institute Finland (Luke), Turku, Finland; 16 State Key Laboratory of Regional and Urban Ecology, Ningbo Observation and Research Station, Fujian Key Laboratory of Watershed Ecology, Institute of Urban Environment, Chinese Academy of Sciences 85406 , Xiamen, China; 17 Zhejiang Key Laboratory of Urban Environmental Processes and Pollution Control, CASHaixi Industrial Technology Innovation Center in Beilun, Ningbo, China; 18 University of Chinese Academy of Sciences, Beijing, China; 19 College of Science and Engineering, Flinders University, Bedford Park, Australia; 20 The Aerobiome Innovation and Research Hub, Flinders University, Bedford Park, Australia; University of Wisconsin-Madison, Madison, Wisconsin, USA

**Keywords:** microbiome, biointegrated design, bioaugmented design, urban ecology, biodesign

## Abstract

Urbanization is accelerating at an unprecedented pace, with 70% of the global population projected to live in cities by 2050. This shift presents significant challenges and opportunities for fostering sustainable urban ecosystems aligned with the United Nations Sustainable Development Goals. Microbiomes—the diverse communities of microorganisms that underpin ecosystem function—are increasingly recognized for their vital role in nutrient cycling, climate regulation, biodiversity support, and human well-being. However, their consideration and integration in urban design remain underexplored, often limited to disease mitigation. The emerging field of microbiome-integrated urban design seeks to leverage microbial activity to enhance urban health and resilience through a multispecies framework. To address critical gaps, the Probiotic Cities Working Group convened a global interdisciplinary workshop, engaging experts from ecology, architecture, urban planning, immunology, and social sciences. Using reverse brainstorming and thematic analysis, participants identified eight core themes and 40 priority research questions (via a modified Delphi technique). These themes span communication and policy, pollution prevention, interdisciplinary collaboration, experimental design, ethics, and public perception of microbiomes. A binomial concordance analysis revealed strong consensus on the top-ranked questions, which address urgent needs such as improving science communication, defining success metrics, and promoting evidence-based microbiome interventions. This paper discusses the top-ranked priority research questions and their broader implications for microbiome science, urban health, and sustainable development. By focusing on these priorities, researchers, policymakers, and practitioners can foster a transformative agenda to integrate microbiomes into urban design, advancing resilient and equitable cities for the future.

## INTRODUCTION

Seventy percent of the world’s population will be urbanized by 2050 (1). The rapid urbanization of the 21st century presents both opportunities and challenges for sustainable development. Urban areas are focal points for addressing global health and are drivers of the biodiversity and climate crises. Aligned with the United Nations Sustainable Development Goals (SDGs), particularly SDG 3 (Good Health and Wellbeing), SDG 11 (Sustainable Cities and Communities), and SDG 15 (Life on Land), cities must evolve in ways that promote human and ecosystem health (2). However, when it comes to designing cities, the focus has been on the visible constituents (e.g., gray and green infrastructure), with limited consideration—aside from disease control—for the microbial domain.

Urban ecosystems support a dynamic microbiome—the collection of bacteria, fungi, and other microorganisms and their theater of activity (3). Microorganisms are omnipresent; they exist on surfaces, in the air, and across natural and built environments (including in and on their resident organisms) (4). Microorganisms play vital roles in nutrient cycling (5), climate regulation (6), food web dynamics (7), animal and plant health (8), and soil formation (9), among many other functions and processes—they are “the glue that holds our ecosystems together.” While research increasingly emphasizes the environmental microbiome’s influence on human health (10, 11) and ecosystem resilience (12), integrating microbiome science into urban design and architecture remains underdeveloped. Cities represent a unique interface of human activity and environmental complexity, where microbial interactions can both support and threaten urban health and ecosystem services (13). There is also growing interest in bio-integrated or augmented design—the incorporation of living organisms, typically microorganisms, into the design and function of anthropogenic systems. Bio-integrated design aims to enhance a system’s sustainability or functionality by leveraging the biological activities of selected organisms (14). Despite growing interest, fundamental gaps remain in understanding how we can systematically foster beneficial microbial environments in urban spaces. Exploring case studies on innovative microbiome monitoring, which identify both beneficial and adverse microbiome environments, and integrating this knowledge with related detection methods can provide practical insights and data to bridge these gaps.

To provide theoretical grounding for this emerging field, we also draw on principles of Responsible Research and Innovation (RRI) (15). RRI emphasizes anticipatory thinking, inclusivity, reflexivity, and responsiveness—principles that align strongly with the goals of microbiome-integrated design. These include involving diverse voices, promoting interdisciplinarity, engaging publics in decision-making, and considering long-term consequences of urban interventions. Such a framing connects our agenda with broader international efforts such as the National Academies’ report on Microbiomes of the Built Environment (16), which similarly calls for integration across disciplines and sectors. Embedding RRI principles in this research agenda ensures that microbiome-integrated design is scientifically robust, socially legitimate, and ethically informed.

To address this gap, the Probiotic Cities Working Group convened a workshop focused on identifying key research questions at the intersection of microbiome science and urban design. This workshop brought together an interdisciplinary team of experts, including ecologists, urban designers, microbiologists, public health researchers, immunologists, and social scientists from across the world, to collaboratively develop a framework and list of priority questions. These questions aim to guide researchers and practitioners toward actionable insights for microbiome-integrated design and to establish a foundation for a resilient and health-oriented urban future. This review, therefore, aims to synthesize current knowledge, identify priority research questions, and provide a structured framework for advancing microbiome-integrated urban design.

This paper presents eight priority themes and 40 priority research questions identified in the workshop and associated processes. Each question offers a pathway for future research projects and other initiatives that advance microbiome-integrated urban design. The aim is to catalyze a new research agenda that aligns the microbiome with sustainable, inclusive, and resilient urban development and embodies RRI principles to ensure this agenda is anticipatory, reflexive, and societally responsive.

## METHODS

**Fig 1:**
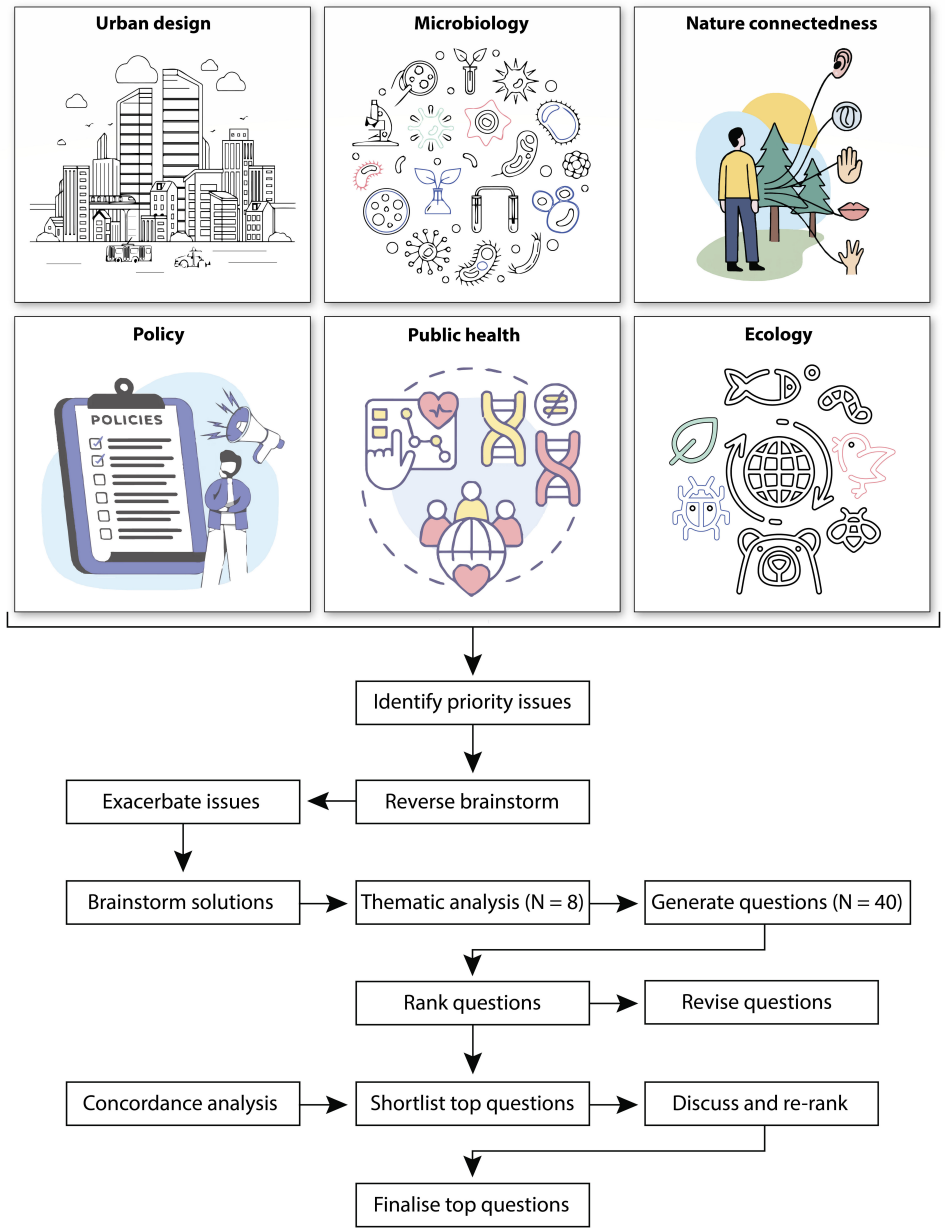
Disciplines involved in the workshop and a workflow showing the process of generating the priority research questions.

The Probiotic Cities Working Group held an in-person workshop on 17 October 2024, at University College London, UK. Invitations to participate were distributed by email and advertised via social media platforms (e.g., X and LinkedIn). The final list of attendees represented interdisciplinary research and practice (in architecture and urban design, public health, ecology, policy, nature connectedness, and microbiology; Fig. 1) and different nationalities, cultural groups, and genders. A total of 18 participants from 10 institutions worldwide (e.g., China, Germany, Australia, Saudi Arabia, Italy, Canada, Finland, UK) made important contributions to the development of the 40 questions presented in this paper, which used a modified Delphi technique (Fig. 1). All contributors are listed as authors.

During the workshop, participants took part in a reverse brainstorming activity. This is a creative problem-solving technique where, instead of seeking solutions to a problem, participants focus on generating ideas to make the problem worse (17). By intentionally brainstorming ways to exacerbate the issue, individuals can identify potential pitfalls, obstacles, and negative factors that might not be immediately obvious. This approach can lead to a deeper understanding of the problem and highlight areas that need attention. Once these detrimental ideas are listed, the process is reversed to find constructive solutions by addressing and mitigating the identified negative factors. Reverse brainstorming encourages lateral thinking and can uncover innovative solutions that might otherwise be overlooked (18). The reverse brainstorming activity aimed to identify key factors that could inhibit the development of microbiome-integrated urban design and co-design potential solutions (Table S1).

The results from the reverse brainstorming session were subsequently analyzed thematically by translating the workshop output and analyzing the script in NVivo—a qualitative data analysis computer software package produced by Lumivero (19).

Qualitative coding was conducted by two members of the author team with prior experience in thematic analysis (J. M. Robinson and R. Beckett). These researchers initially coded the transcripts independently, then met to compare coding approaches and resolve any discrepancies through discussion. This reflexive process ensured consistency in how codes were applied. Following this, all co-authors were invited to review the emerging codebook and provide feedback, allowing for minor refinements. The translation of workshop outputs into the 40 preliminary research questions was carried out collaboratively: the two initial coders drafted the questions based on the eight common themes, which were then refined in consultation with the broader author group to ensure accuracy and inclusivity of perspectives.

Once the results were thematically analyzed, a set of 40 preliminary research questions was produced to align with eight common themes. These preliminary questions were then distributed to all Probiotic Cities Working Group members (including all workshop participants). All 18 participants took part in the ranking task. Participants were then asked to assign each question a score of 1–5 (1 = top priority, 5 = bottom priority), depending on their potential importance and impact and the presence of considerable knowledge gaps (20). All questions were retained, but only the top-ranked questions (i.e., one from each theme) would be highlighted and discussed further in this paper. To determine agreement among contributors, we ran a Kendall’s coefficient of concordance and a binomial test in Python to count the number of times the top question was ranked as such and to determine if this proportion is significantly higher than random chance (e.g., 1/5 = 20% for five questions). All top-ranked questions with a *P*-value of ≤0.05 were automatically retained. Any with a *P*-value of >0.05 were redistributed to the contributors for further discussion along with the second-ranked question in that theme. During this second round, participants were asked to provide a short justification if they wished to “upgrade” or “downgrade” a question. These justifications were reviewed collectively during group discussions by email to ensure a transparent and democratic process. At this point, the remaining questions could be upgraded or downgraded. A thorough and valid justification for any upgrades or downgrades was provided and agreed upon, as required. To ensure a democratic process was upheld, participants were asked to confirm support or otherwise for any decision before finalizing the 40 priority questions.

## REVERSE BRAINSTORMING RESULTS

**Fig 2:**
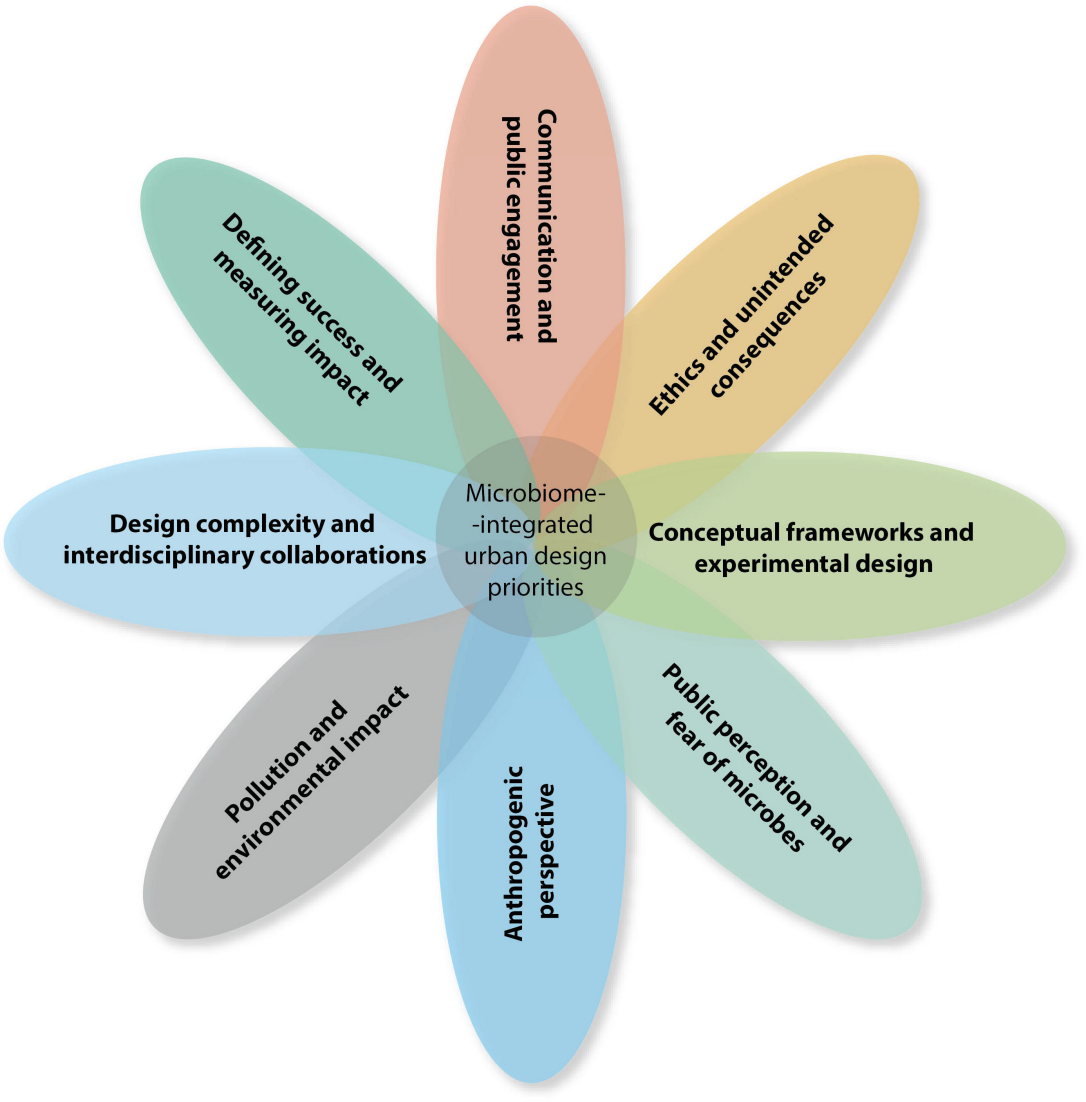
Venn diagram showing the eight interconnected microbiome-integrated urban design research themes identified during the thematic analysis.

A summary of the reverse brainstorming results is listed in Table S1. Following thematic analysis, we identified eight core themes (Fig. 2), including Theme 1: Communication and public engagement, Theme 2: Defining success and measuring impact, Theme 3: Design complexity and interdisciplinary collaborations, Theme 4: Pollution and environmental impact, Theme 5: Conceptual frameworks and experimental design, Theme 6: Anthropogenic perspective, Theme 7: Ethics and unintended consequences, and Theme 8: Public perception and fear of microbes.

Within each theme, we identified five priority research questions, for a total of 40 included questions across all themes. The top-ranked question from each theme is listed in Table 1. Our binomial concordance analysis revealed that five out of eight top-ranked questions had *P*-values of <0.05, meaning that there is strong statistical evidence that these questions were ranked first more often than expected by random chance and received or approached a voting majority. These questions were immediately retained for discussion. Three questions (from Themes 4, 5, and 8) were returned to the contributors for further scrutiny. These questions received the highest overall ranking for their respective themes, but only moderate concordance among the multiple contributors. The remaining 32 priority questions can be found in Table 2, and a word cloud of reverse brainstorming common terms is in Fig. S1.

**TABLE 1:** Top-ranked priority research questions

Theme	Question	Mean	SD	% (rank 1)	Binomial *P*-value
1	How can we improve science communication to make microbiome research accessible to policymakers?	1.73	1.29	73%	<0.001^*a*^
2	What methods/metrics best capture the long-term success of microbiome interventions in urban settings?	1.73	0.92	53%	0.004^*a*^
3	How does the inclusion of multiple disciplines impact the efficacy and scalability of microbiome-based urban design?	2.00	1.21	47%	0.018^*a*^
4	What role can microbiomes play in promoting a circular economy and pollution prevention?	2.00	1.10	40%	0.061
5	What standards can be set for creating reliable, repeatable experiments in microbiome-integrated urban design?	2.27	1.24	40%	0.061
6	How effective are early childhood interventions, such as forest schools, in fostering respect and awe for microbiomes?	2.00	1.34	47%	0.018^*a*^
7	What are the best practices for implementing long-term, evidence-based microbiome interventions?	1.87	0.88	47%	0.050^*a*^
8	What strategies can be used to promote the positive benefits of microbiomes to the public?	2.07	0.93	33%	0.164

^*a*^ Significant.

**TABLE 2:** All themes and priority questions

Theme	Rank	Question
1: Communication and public engagement	1	How can we improve science communication to make microbiome research accessible to policymakers?
	2	What visual communication techniques can most effectively enhance public understanding of microbiome-integrated design?
	3	What are the impacts of community-led labs on public engagement with microbiome science?
	4	To what extent does teacher training in microbiome science influence science literacy and acceptance in communities?
	5	What are effective methods to translate technical jargon into accessible language for the public?
2: Defining success and measuring impact	1	What methods/metrics best capture the long-term success of microbiome interventions in urban settings?
	2	What standardized methods can be developed to measure the ecological, economic, and social success of microbiome-integrated urban design projects?
	3	How can we co-develop visions of success with stakeholders for microbiome-based interventions?
	4	What frameworks are most effective for establishing long-term roadmaps and milestones for microbiome-integrated design projects?
	5	What role does demystifying microbiome science play in setting clear project goals?
3: Design complexity and interdisciplinary collaboration	1	How does the inclusion of multiple disciplines impact the efficacy and scalability of microbiome-based urban design?
	2	How can interdisciplinary collaborations reduce design complexity in microbiome-integrated projects?
	3	What decision support systems can be used to simplify complex design parameters in microbiome projects?
	4	What are the core design constraints needed for microbiome-integrated urban projects?
	5	How can incremental learning processes (for researchers and designers) improve the design of microbiome interventions?
4: Pollution and environmental impact	1	What role can microbiomes play in promoting a circular economy and pollution prevention?
	2	How do feedback loops between microbes and pollutants affect urban ecosystems?
	3	What frameworks can be developed to measure the effectiveness of microbes in mitigating urban pollution?
	4	How can real-time pollution monitoring help design better microbiome-integrated solutions?
	5	How does pollution from other industries impact the implementation of microbiome-based interventions?
5: Conceptual frameworks and experimental design	1	What standards can be set for creating reliable, repeatable experiments in microbiome-integrated urban design?
	2	What are the key components of a conceptual framework for microbiome-integrated design?
	3	How can outdoor living labs contribute to robust, replicable methodologies in microbiome science?
	4	What role do randomized controlled trials (RCTs) play in evaluating microbiome-based interventions?
	5	How can poor experimental design be mitigated in interdisciplinary microbiome research?
6: Anthropogenic perspectives	1	How effective are early childhood interventions, such as forest schools, in fostering respect and awe for microbiomes?
	2	How can multi-species education promote ecosystem thinking in urban communities?
	3	What are the effects of anthropocentric perspectives on the adoption and efficacy of microbiome-based urban projects?
	4	How can systems thinking frameworks incorporate non-human stakeholders in urban design?
	5	What role do companion species play in promoting awareness of microbial ecosystems?
7: Ethics and unintended consequences	1	What are the best practices for implementing long-term, evidence-based microbiome interventions?
	2	How can frameworks for the ethical use of synthetic communities in microbiome research be created?
	3	What are the risks of invasive species with microbiome-based synthetic communities?
	4	How can project phasing help mitigate unintended consequences in microbiome interventions?
	5	How can ethical considerations be balanced with the speed of innovation in microbiome-integrated design?
8: Public perception and fear of microbes	1	What strategies can be used to promote the positive benefits of microbiomes to the public?
	2	How can educational initiatives reduce fear and negativity towards microbes in urban populations?
	3	How does nature connectedness influence public attitudes towards microbes?
	4	What are the impacts of immersive, engaging forms of research translation on public understanding of microbes?
	5	How can the public perception of microbes be shifted away from a pandemic-only focus?

## THE TOP-RANKED PRIORITY RESEARCH QUESTIONS

Here, we highlight and discuss the top-ranked priority research questions in microbiome-integrated urban design, as identified through our analysis. These questions represent areas of shared interest and urgency among respondents, reflecting the collective perspective of stakeholders in this emerging field. Focusing on these top priorities, we aim to explore their broader implications for advancing microbiome science and its integration into urban ecosystems. Furthermore, these questions provide critical insight into the gaps in current knowledge, practical challenges, and opportunities for impactful inter- and trans-disciplinary collaboration. By addressing these priority areas, researchers, policymakers, and practitioners can drive meaningful progress in creating healthier, more sustainable, and resilient urban environments that incorporate the vital role of microbiomes.

### Theme 1: Communication and public engagement

#### How can we improve science communication to make microbiome research accessible to policymakers?

This question underscores the need to bridge the gap between scientific research and policy implementation, broadening political discussion in the field of environmental microbiology. The question scored highly in our concordance analysis (X̄ = 1.73, SD = 1.29). Consideration of the microbiome in policy is limited. Moreover, traditional means of communicating scientific evidence to policymakers, such as POSTNotes in the UK, focus on a narrow range of microbe-related themes (e.g., medical, food, soil function, applications in waste management). There is also limited research on the ability of policymakers to engage with and understand the microbiome. Accessible communication of microbiome science to policymakers is crucial for fostering evidence-based decision-making in urban planning and public health. By translating often complex findings into actionable insights, addressing this question can enhance policy support for microbiome-integrated urban design, enabling solutions that improve ecosystem health and urban resilience. One such example is the Microbiome-Inspired Green Infrastructure framework (21, 22), which was developed to bridge the disciplines of microbial ecology and landscape architecture and to translate complex concepts into intelligible formats for policymakers. This framework has been used by urban developers (e.g., Lendlease) to inform salutogenic urban designs. Collaborating with artists to make the “invisible visible” could also bring immense value to this priority (23, 24). Artistic interpretations can visualize microbial processes and ecosystems, helping policymakers and the public better understand and empathize with the unseen, including the crucial roles microbiomes play in urban health. There is growing evidence that science-art collaborations can stimulate emotional engagement, spark dialog, and create accessible entry points into complex scientific issues. For example, Bencard and Whiteley (23) describe how sci-art projects in biomedical research environments increased reflexivity and broadened discussions among both researchers and external stakeholders, while Greenhough et al. (24) show how visual and participatory methods can open up new forms of engagement around environmental science. Bio-art installations and interactive exhibitions, such as those by artist Heather Barnett, have effectively communicated the beauty and complexity of microbial networks. Although formal evaluations of policymaker responses to sci-art are still limited, these projects demonstrate that art-science collaborations can draw policymakers into conversations they might not otherwise engage with, by combining intellectual commentary with immersive, affective experiences.

Beyond sci-art, there is also a need for more conventional and targeted science-communication approaches. Evidence shows that policymakers prefer concise, visually clear outputs such as infographics, policy briefs, and 1–2 page summaries (25). Web-based interactives or dashboards that link microbiome data to urban health outcomes could also offer intuitive entry points. Short verbal pitches (e.g., 90 second “elevator talks”) tailored for staffers and advisors may be especially effective, given their role in briefing decision-makers. Engaging science journalists to cover microbiome-urban health linkages is another route to indirectly influence policy agendas. It is also important to consider scale: local and municipal policymakers (e.g., city planners, regulatory authorities) often make decisions directly relevant to urban design standards, while national or federal policymakers can influence funding, regulation, and strategic priorities. Targeted engagement with specific policymakers who have decision-making authority in public health, architecture, and environmental regulation may therefore be more impactful than generic outreach to “all policymakers.”

Collaborations such as the Microbial Childhood Collaboratory (https://research.tuni.fi/ecepp/microbial-childhood-collaboratory-mcc/) offer a practical example of this approach, integrating community art with expertise from diverse research fields. While empirical evidence of direct policy impacts is still emerging, such initiatives show promise in creating new interfaces where policymakers, practitioners, and publics can encounter microbiome-related issues in more tangible and relatable ways. These collaborations could inspire emotional and intellectual connections with microbiome science, encouraging greater interest and investment in microbiome-informed urban planning. By fostering creativity and accessibility, such interdisciplinary efforts can amplify the reach and impact of microbiome research.

### Theme 2: Defining success and measuring impact

#### What methods/metrics best capture the long-term success of microbiome interventions in urban settings?

The long-term impact of microbiome interventions requires robust, standardized metrics to evaluate their ecological and social benefits comprehensively. These metrics are essential to understanding how microbiome-based strategies perform over time, particularly in complex urban ecosystems where multiple variables interact. The question also scored highly in our concordance analysis (X̄ = 1.73, SD = 0.92), reflecting its perceived importance among experts. Developing these metrics is critical for creating a framework that can guide future projects by identifying clear indicators of success and providing benchmarks for comparison across different contexts and regions. Standardized measures allow for consistent evaluation of key outcomes, such as biodiversity enhancement, improvements in air and soil quality, reductions in environmental pollutants, and advancements in public health metrics. Such evaluations are not only necessary for assessing the ecological efficacy of microbiome interventions but also for demonstrating their value to policymakers, urban planners, and the broader public. Moreover, these metrics support the monitoring of sustainability goals by tracking progress over time and ensuring that interventions align with broader objectives, such as enhancing urban resilience to climate change and improving community well-being. For example, integrating microbial indices (including composition and functional roles) with urban air quality monitoring systems could provide a more holistic understanding of how microbiome interventions contribute to healthier urban environments. Recent research has begun to propose such integrative approaches, for instance, by combining ecological indicators with microbial community assessments to monitor restoration outcomes (e.g., references 10, 12). Case study methodologies are also increasingly recognized as essential for practical validation, allowing researchers to test frameworks in real-world contexts and refine metrics based on observed outcomes (e.g., references 26, 27).

In addition, observational studies have provided critical insights into how microbiomes mediate links between environment and health. For example, Amato et al. (28) review how human health outcomes are shaped by microbiome variation and how social and built environments influence these dynamics (28). Complementarily, interventional studies have tested microbiome-based strategies to improve health, with Fujimura et al. (29) showing how controlled microbial exposures can modulate immune function and resilience (29). These approaches highlight the value of incorporating both observational and experimental data into the development of robust metrics for urban microbiome interventions.

Lessons can also be drawn from other domains where multiple data streams are integrated to assess the “health” of built environments. In hospitals, for example, researchers have monitored microbial community composition alongside patient infection rates, antimicrobial resistance (AMR) profiles, and cleaning protocols to better understand links between the built microbiome and health outcomes (30, 31). Similarly, in agriculture and food safety, hazard analysis and critical control point frameworks incorporate microbial surveillance, hygiene practices, and product testing to manage microbial risk (32). These examples illustrate that combining microbial, environmental, and human health metrics provides a richer understanding of system performance, and urban microbiome interventions may benefit from adapting similar multi-metric frameworks. Exploring these cross-sectoral examples highlights the potential of borrowing methods from healthcare and food industries to inform urban contexts. In doing so, researchers and practitioners can move toward hybrid frameworks that capture microbial dynamics, but also social, ecological, and health-related dimensions of intervention success.

The importance of this question is underscored by its urgency; developing such a metric takes time and resources. However, by addressing this question, researchers and practitioners can ensure that microbiome-based solutions deliver measurable and meaningful benefits for ecosystems and society.

### Theme 3: Design complexity and interdisciplinary collaborations

#### How does including multiple disciplines impact the efficacy and scalability of microbiome-based urban design?

Microbiome-integrated urban design is inherently interdisciplinary, requiring input from ecologists, architects, urban planners, and social scientists. Exploring this question highlights the importance of cross-disciplinary collaboration for overcoming design complexity, improving scalability, and ensuring interventions are innovative, grounded in diverse expertise, and impactful. This approach can unlock new strategies for tackling urban ecosystem challenges. The “Biophilic Streets” design framework illustrates this by combining urban planning, ecology, and public health to create streetscapes that enhance biodiversity and human well-being (33). Case studies demonstrate that such collaborative approaches can lead to scalable and effective urban designs that incorporate microbiome considerations.

Additionally, engaging with creative disciplines and storytelling can deepen public and stakeholder engagement with microbiome-based urban design. For example, in the “Living Buildings” project, architects and microbiologists collaborated to incorporate living microbial surfaces into architectural spaces, blending functional design with microbial ecology (34). This approach showcased the aesthetic and ecological potential of microbiome-integrated solutions while fostering public appreciation for the hidden world of microbes. Additionally, interdisciplinary collaborations help standardize methodologies, ensuring that microbiome-integrated projects are scalable, relevant, and replicable across different urban and cultural contexts. By leveraging the strengths of multiple disciplines, microbiome-based urban design can develop innovative, evidence-based interventions that address the complex interplay of environmental and human health factors in urban ecosystems.

### Theme 4: Pollution and environmental impact

#### What role can microbiomes play in promoting a circular economy and pollution prevention?

Microbiomes have significant potential to recycle waste, degrade pollutants, and support sustainable resource use in cities. This question addresses their role in creating circular economies, where waste becomes a resource, thereby reducing environmental footprints and enhancing urban ecosystem health. The area receiving the most attention in this field utilizes microbes to reduce plastic pollution and enable upcycling of plastics into high-value chemicals (35). Foundational microbial ecology research has long documented microbial pathways for biodegradation, from hydrocarbon-degrading bacteria in oil spill clean-up (36) to lignocellulose breakdown in soils and compost (37). These classical studies demonstrate the metabolic versatility of microbes and their capacity to transform waste into usable products, forming the scientific basis for current applications in urban circular economies.

Recent case studies highlight this translational potential. For example, *Ideonella sakaiensis* and related taxa have been shown to depolymerize polyethylene terephthalate (PET) plastics under relatively mild conditions (38), and microbial consortia have been trialed in pilot-scale bioreactors to convert plastic-derived monomers into bioplastics or chemical feedstocks (39). Similarly, microbial fuel cells have been deployed in wastewater treatment facilities to simultaneously degrade organic waste and generate electricity (40), illustrating how microbial processes can close resource loops in urban systems.

But there is much potential for broadening the scope of microbially facilitated circular economies. Advancing research in this area could improve urban sustainability practices. The aerobiome is a growing area of research (41). Historically, bioaerosol research has focused on preventing diseases by controlling pathogens and understanding the impacts of airborne pollutants. However, an emerging paradigm shift in thinking (42) reframes bioaerosols as not just threats but valuable components of ecological and urban systems. The aerobiome likely affects pollution dynamics (and vice versa) (43), influencing production of and exposure to pollutants. For instance, studies have shown that airborne microbial communities can interact with atmospheric particulates, altering their chemical composition and deposition rates (44), suggesting overlooked pathways linking air microbiology and pollution cycles.

Airborne microbes also play vital roles in food systems (45), and these have yet to be investigated in relation to developing circular economies. Furthermore, a circular economy is premised on the longevity and durability of materials, which the microbiome also influences. In materials science, an understanding of the microbiome has enabled researchers to evaluate which microbes decay ancient monuments most quickly, under which conditions, and how to apply different microbes to enhance the preservation of building materials (46). Conservation microbiology has already applied such knowledge, using microbial bio-consolidation techniques to protect stone heritage sites from weathering (47), providing a model for future applications in urban construction and maintenance.

In urban green infrastructure, longevity with minimal maintenance is often taken as a given, but these systems, which include street trees, green roofs, and living walls, are vulnerable to poor ecological design, poor maintenance, and climate change, increasing the need to replace materials and organisms over time. Recent research highlights the importance of microbes in enhancing the longevity of plants within these systems (e.g., green roofs) (48), which in themselves contribute to pollution control and circular, resilient cities (49, 50). Further research in these areas is important.

### Theme 5: Conceptual frameworks and experimental design

#### What standards can be set for creating reliable, repeatable experiments in microbiome-integrated urban design?

Reliable experimental frameworks are essential for advancing microbiome science. Setting standards ensures research is reproducible and outcomes are comparable across different contexts. This question is important for establishing credibility in the field and facilitating the translation of research findings into real-world urban solutions. For example, global initiatives led by the Earth Microbiome Project (51) and MetaSUB Consortium (Metagenomics and Metadesign of Subways and Urban Biomes) (52) have developed standardized sampling protocols that are now being adapted for urban microbiome research to ensure data comparability across cities. Concrete examples of applying such frameworks already exist. For instance, Kembel et al. (53) used standardized high-throughput sequencing of airborne microbes across buildings to reveal how design factors such as ventilation systems shape microbial communities, providing a model for how sampling strategies can be integrated into architectural and environmental research. Similarly, Roslund et al. (54) applied experimental protocols to green-space interventions in childcare centers, demonstrating how manipulations of forest floor biodiversity can improve children’s immune regulation. These studies exemplify how robust methodological design can link microbiome science directly to outcomes relevant for human health and urban sustainability.

**Fig 3:**
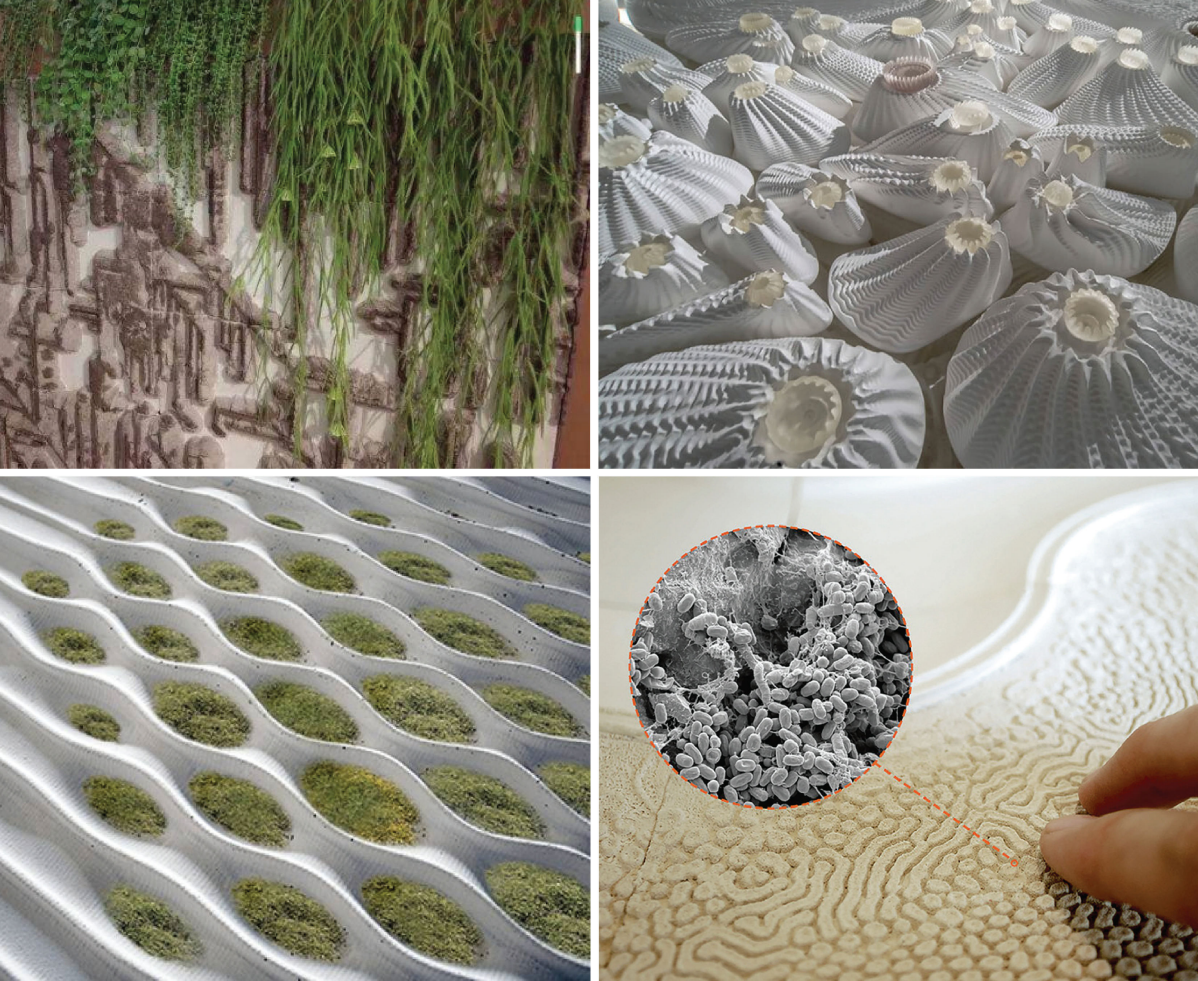
Examples of indoor bio-augmented materials that integrate living or microbial-compatible systems into design. Top left: bio-integrated wall system combining structural substrate with cascading vegetation to support plant-microbiome interactions indoors. Top right: 3D-printed modular structures inspired by natural forms (e.g., coral and fungi), designed as porous habitats that can host microbial and plant life. Bottom left: experimental wall cladding with patterned micro-reservoirs for moss and microbial colonization, enabling living green surfaces in controlled indoor environments. Bottom right: close-up of a bio-textile surface designed for tactile interaction, with inset scanning electron micrograph showing microbial colonization at the microscale (of the beneficial *Bacillus subtilis*).

However, broader development is required to adapt these frameworks for microbiome-integrated urban design projects that extend beyond research into application. For instance, designing urban green spaces or indoor bio-augmented materials (Fig. 3) that optimize salutogenic microbial exposures requires experimental standards that account for local ecological variables, human interactions, and long-term impacts. Expanding and refining these frameworks will enable researchers and practitioners to implement and evaluate interventions more effectively, ultimately promoting healthier, more sustainable urban ecosystems.

In addition to research-led standards, a partnership with practice-focused standard-setters could accelerate translation. The International WELL Building Institute (IWBI) develops and disseminates standards for human health and well-being in buildings and has an active research program aimed at evidence-based criteria. Collaborating with IWBI could help articulate what constitutes a “healthy urban-space microbiome” in ways actionable for designers, developers, and regulators. Concretely, this might include (i) pilot credits or an addendum specifying microbiome sampling design (spatial/temporal replication, controls), (ii) minimum metadata and QA/QC requirements (e.g., surface/air/soil matrices, occupancy patterns, cleaning regimes), (iii) reporting conventions and performance indicators (e.g., community diversity/evenness, persistence of sentinel taxa, AMR risk profiling), (iv) commissioning and re-commissioning checks for bio-augmented materials and green infrastructure, and (v) longitudinal monitoring and public reporting to support continuous improvement. Positioning such criteria within a recognized market standard (e.g., via IWBI guidance) would complement open scientific frameworks (e.g., Earth Microbiome, MetaSUB), reduce heterogeneity in methods across projects, and provide clear targets for procurement and policy. This dual-track approach—open science protocols plus practice standards—can bridge the gap between experimental design and scalable, verifiable implementation in real urban settings.

### Theme 6: Anthropogenic perspective

#### How effective are early childhood interventions, such as forest schools, in fostering respect and awe for microbiomes?

This question highlights the role of education in shaping future generations’ understanding of microbiome-integrated design and its potential to promote healthier, more sustainable urban ecosystems. Connection to nature is one of the strongest predictors of pro-environmental behaviors, and research suggests that this connection is most likely to be fostered in childhood (55, 56). The microbiome rarely features in our conception of “nature,” but could contribute to these long-term behavioral changes and environmental stewardship. For example, a program in Finland introduced diverse microbial communities via local forest floor materials into outdoor education center environments, leading to measurable improvements in children’s microbiomes and immunoregulation (54). These interventions also align well with the nature connectedness question (see Theme 8, question 2) (55). Forming bonds with (the rest of) nature is a key driver of long-term engagement with nature conservation and can support the broader goals of sustainable urban design. By integrating microbiome science and related creative activities into educational settings, we can create opportunities to embed nature connectedness and shape new environmental values (considering the invisible biodiversity) in future generations (57).

### Theme 7: Ethics and unintended consequences

#### What are the best practices for implementing long-term, evidence-based microbiome interventions?

Long-term microbiome interventions carry ethical and ecological implications that require careful consideration. This question addresses the need for practices that ensure interventions are sustainable, are socially acceptable, and minimize unintended ecological consequences. There are growing concerns about microbial technology from a personal and ecological perspective (58–60). For instance, microbial inoculants modify native soil communities and functions through competition, antagonism, synergism, and changed root exudation (58). Though research surrounding the impact of microbial inoculants is in its infancy, we know that modified microbial communities through other mechanisms, such as the introduction of invasive species, have detrimental impacts on native biodiversity (61). However, we have not fully assessed the potential long-term and multidimensional impacts on natural systems. Developing evidence-based guidelines will build trust and foster responsible innovation in urban microbiome applications.

The field of microbiome-integrated urban design can also learn from other emerging technologies that have developed frameworks to build trust and foster RRI. For example, Stilgoe et al. (15) outline core principles of RRI—anticipation, inclusivity, reflexivity, and responsiveness—that can guide the governance of novel interventions (15). Similarly, in the context of gene drive and genetic engineering, Long et al. (62) proposed “core commitments” for field trials, including transparency, community engagement, risk assessment, and adaptive governance. These precedents show how structured ethical commitments and stakeholder-inclusive processes can help navigate uncertainty, ensure accountability, and address public concerns.

Adapting such principles to microbiome interventions would involve developing transparent reporting frameworks, participatory consultation processes with communities and policymakers, and robust monitoring programs to detect unintended ecological shifts. Drawing on these prior RRI-informed approaches could accelerate the responsible scaling of microbiome interventions, ensuring they are both scientifically robust and societally legitimate.

### Theme 8: Public perception and fear of microbes

#### What strategies can be used to promote the positive benefits of microbiomes to the public?

Public understanding of microbiomes often focuses on negative aspects, such as disease. This question seeks to shift perceptions by emphasizing the benefits of microbiomes in improving urban ecosystems and public health. General microbial literacy is low in the population, and this is associated with a fear of microbes and “dirt,” also known as “germaphobia” (63). Effective strategies could include education campaigns, community-led projects, and accessible communication to build awareness and acceptance of microbiome-integrated solutions. The “Designing the Urban Microbiome” initiative explores how integrating microbial considerations into urban design can improve public health and environmental quality (64). By engaging the public through design and architecture, this approach fosters a positive understanding of microbiomes in urban settings. Professor Kenneth Timmis started an initiative called the International Microbiology Literacy Initiative (https://imili.org/) to emphasize the need for microbiology literacy (65). This initiative aims to balance the public’s perception of microbes and draw attention to the multitude of benefits they bring (in addition to the pathogenic aspects). More of these initiatives should be a priority.

At the same time, engagement strategies must avoid persuasion-based or “deficit” models of communication. Many people’s lived experience of microbes is framed by illness, infection control, and hygiene practices, so concerns about risks and unintended consequences of microbiome-based interventions are legitimate and must be taken seriously. Following RRI principles (15), engagement should emphasize inclusivity, responsiveness, and reflexivity, ensuring that diverse publics have the opportunity to articulate questions, values, and concerns. This requires dialogue-based approaches such as citizen forums, deliberative workshops, and participatory design processes, where publics can co-shape priorities and contribute to decisions about microbiome integration in urban spaces.

Acknowledging risks (e.g., potential pathogenicity, unintended ecological effects) alongside benefits is critical for building trust. Transparent discussion of trade-offs and uncertainties, coupled with community participation in both the framing of research questions and the evaluation of interventions, can help establish legitimacy and public ownership. Rather than being framed as simply “educating” the public, microbiome communication should instead be positioned as a two-way process that builds literacy while also valuing and incorporating societal perspectives.

## POLICY RECOMMENDATIONS

To accelerate the integration of microbiome-integrated thinking into urban design, the priority is to (i) develop step-by-step guidelines for integrating microbiome science into urban policy, including establishing cross-disciplinary advisory panels, funding pilot projects, and developing regulations for microbial interventions; (ii) create standardized metrics and methodologies to evaluate the long-term success of microbiome interventions, including robust experimental frameworks and monitoring systems; and (iii) develop effective strategies to educate the public and policymakers about the benefits of microbiomes in improving urban ecosystems and public health, including visual campaigns, community-driven projects, and workshops.

In strengthening these recommendations, lessons can be drawn from policy work on other emerging technologies. For example, Long et al. (62) proposed a set of “core commitments” for gene drive field trials, including transparency, engagement, risk assessment, and adaptive governance, which are equally relevant for microbiome-based interventions. More broadly, the RRI literature (15) provides principles—anticipation, inclusivity, reflexivity, and responsiveness—that have informed policy frameworks for biotechnology, nanotechnology, and synthetic biology. These precedents show that clear governance structures, inclusive stakeholder processes, and iterative monitoring mechanisms can help manage uncertainty and build trust as microbiome-based urban design moves from concept to practice. Embedding our recommendations within this wider body of emerging-technology policy scholarship not only bolsters their credibility but also provides a roadmap for ensuring that microbiome integration is scientifically robust, ethically sound, and socially legitimate.

## CONCLUSION

As the world becomes increasingly urbanized, integrating microbiome science into urban design presents an unparalleled opportunity to address some pressing challenges of the 21st century, including biodiversity loss, climate change, and public health crises. This paper identifies eight key themes and priority research questions that serve as a roadmap for advancing microbiome-integrated urban design. These questions underscore the importance of interdisciplinary collaboration, robust experimental frameworks, effective communication, and inclusive approaches that account for both human and ecological health. By fostering cross-disciplinary efforts, microbiome-based urban design can overcome inherent complexities and provide scalable, sustainable solutions. The inclusion of diverse expertise ensures that interventions are not only innovative but also grounded in practical and scientific rigor. Moreover, the research highlights the critical need for standardized metrics and methodologies to evaluate the long-term success of microbiome interventions. Reliable benchmarks are essential for ensuring that these interventions deliver measurable benefits, including enhanced biodiversity, improved air and soil quality, and better public health outcomes. Crucially, this paper emphasizes the importance of public engagement and education across the lifespan to improve microbial literacy and shift perceptions of microbes from fear to appreciation. The future of resilient and equitable cities partially lies in our ability to embrace the unseen yet foundational contributions of microbes. By addressing these priority questions and advancing research in microbiome-integrated urban design, we can build cities that are not only sustainable and inclusive but also better aligned with the complex dynamics of natural systems.

## SUPPLEMENTAL MATERIAL

Supplemental Material msphere.00619-25-s0001.pdf Table S1 and Fig. S1.

ASM does not own the copyrights to Supplemental Material that may be linked to, or accessed through, an article. The authors have granted ASM a non-exclusive, world-wide license to publish the Supplemental Material files. Please contact the corresponding author directly for reuse.
