# Supplementary material for: Priority research questions in microbiome-integrated urban design
Source: mSphere. 2025 Oct 14;10(11):e00619-25. doi: 10.1128/msphere.00619-25 (PMC12645966; doi:10.1128/msphere.00619-25)
Supplement: Supplemental Material — Table S1 and Fig. S1. [file msphere.00619-25-s0001.pdf]

Supplementary Material

Table S1. Results of the reverse brainstorming activity.

Problems	Exacerbated	Solutions
Communication with experts in policymaking.	Overly technical jargon and poor translation. Increase misinformation.	Science communication for education and developing a common language. Visual communication. Proper protocols. Teacher training. ‘Open air’ community-led labs.
No clear idea of what success looks like.	No clear goals. No visioning. Misinformation.	Standard measuring methods. Goals and roadmaps. Co-developing visions. Demystifying
Design complexity	Increase the number of parameters.	Interdisciplinary collaborations. Decision support systems. Incremental learning-design process. Defining design constraints.
Pollution from other industries.	Reducing regulations.	Research on microbe-pollution feedback loops. Circular economy. Pollution prevention.
Lack of conceptual frameworks and core research.	Poor experimental design. Lab-only research.	Outdoor living labs. Replicable methods. Robust interventions and RCT studies.
Anthropogenic views.	Supress free thinking. Reduce rights for non-humans.	Multi-species teaching. Systems thinking. Companion species. Embracing forest schools and early-childhood interventions.
Unintended consequences of synthetic communities.	Increase invasive species. Removal of ethical frameworks. Moving too quickly.	Create ethical frameworks. Long-term studies outdoors. Evidence-based interventions. Project phasing. Clear pathways for implementation.

Public view based on fear and negativity (towards microbes) and nature disconnection.	Reinforce negative perception of microbes. Only link microbes to pandemics.	Translate the research into engaging forms. Educate on the benefits of microbes. Promote nature connectedness activities.
Lack of effective cost analysis.	Don't consider the co-benefits of prioritising costs	Phasing of projects to analyse costs. Holistic assessments of value vs costs.
Institutional resistance.	Propagate conspiracy theories. Highlight failures.	Success stories. Prototyping. Education on microbes in schools. Identifying funding streams.
Lack of funding.	Restrict funding. Increase the use of paywalls.	Build networks and apply for joint funding.
Lack of inter and transdisciplinarity.	Keep working in silos. Increase bureaucratic processes and discourage collaborations.	Promote inter and transdisciplinarity. Global open databases.
Challenges will scalability.	Limit project scale.	Develop easier and cheaper to implement designs and tools.

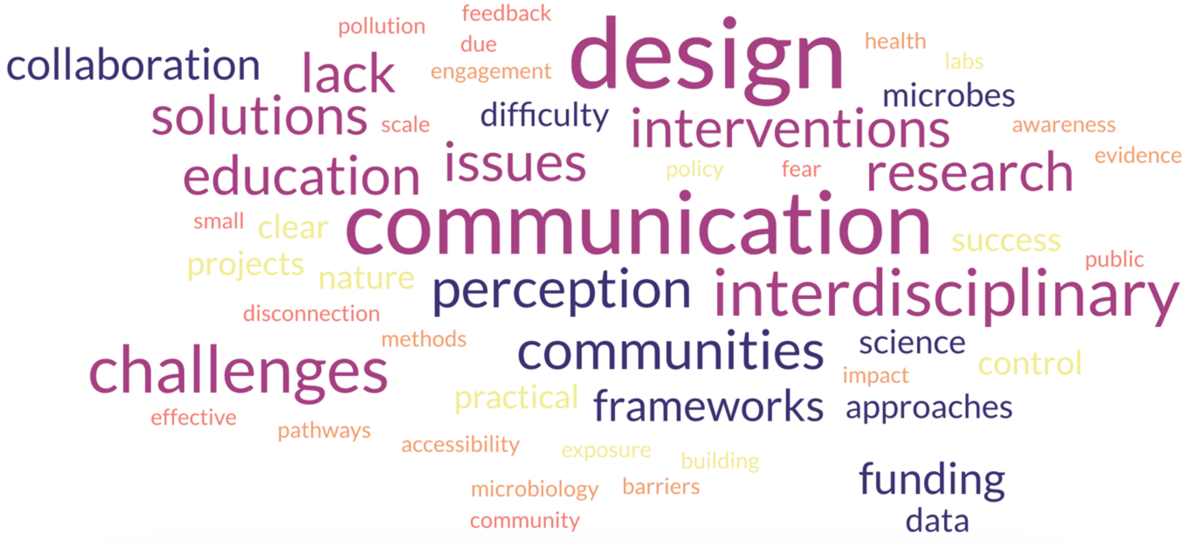

Figure S1. Word cloud of workshop outputs—indicating common terms.
